# Nutrition Literacy: What Are Young Adults With Type-1 Diabetes Missing?

**DOI:** 10.7759/cureus.39899

**Published:** 2023-06-03

**Authors:** Cassandra Abrams, Jaymie Meliker, Anna Floreen Sabino

**Affiliations:** 1 Pediatric Endocrinology, Alabama College of Osteopathic Medicine, Dothan, USA; 2 Family, Population, and Preventive Medicine, Stony Brook University, Stony Brook, USA; 3 Diabetes and Endocrinology, Finding Smiles Coaching, LLC, Boston, USA

**Keywords:** type 1 diabetes mellitus, public health, endocrinology and diabetes, nutrition and metabolism, adolescent and young adult

## Abstract

Introduction: This study evaluated the nutrition literacy and perceived emotional burden of disease in young adults with type-1 diabetes. All participants are current or past members of the non-profit organization The Diabetes Link, formally known as the College Diabetes Network. The Diabetes Link is a 501(c)(3) nonprofit organization working to connect and support young adults with type-1 diabetes through the transitional periods of their lives, most commonly the transition from high school to college. Previous research shows that there is a significant uptick in glycated hemoglobin (HbA1c) levels in people with type-1 diabetes between the ages of 18 and 24, a period associated with many transitional events. While there are numerous hypothesized reasons why HbA1c levels spike during these ages, the lack of nutritional knowledge is frequently highlighted as a root cause of this increase.

Methods: Participants were asked to complete a 40-question survey via Google Forms (Google LLC, Mountain View, California, United States) that contained questions pertaining to their treatment, dietary habits, confidence in healthcare professionals to provide nutrition advice, and overall feelings toward their diagnosis of type-1 diabetes. The survey also included four questions aimed at evaluating the participants' carbohydrate-counting skills to determine a basis of their nutritional knowledge. A binary logistic regression was performed using IBM SPSS Statistics for Windows, Version 27 (Released 2020; IBM Corp., Armonk, New York, United States) to assess the influences of the burden and carbohydrate-counting knowledge on the participants’ diabetes care, eating habits, and emotional outlook on nutrition.

Results: Data from this study show that the participants who scored high on the carbohydrate-counting quiz were 2.389 times more likely to avoid eating because of an out-of-range blood sugar level (p-value = 0.05), and the participants who reported higher levels of burden were 9.325 times more likely to avoid social gatherings because of food (p-value = 0.002).

Conclusion: Results from this study demonstrate that the emotional burden associated with eating and not nutrition knowledge could contribute to the previously listed spike in HbA1c levels.

## Introduction

Currently, 1.25 million Americans live with type-1 diabetes, with projections estimating that this population will reach 5 million by 2050 [1]. The Diabetes Link, formally known as the College Diabetes Network, was founded in 2009 by Christina Roth, who has type-1 diabetes and who felt alone and overwhelmed being a college student with the disease [2]. Today, The Diabetes Link is a 501(c)(3) non-profit organization working under the mission statement of providing young adults with type-1 diabetes with peer connection, resources, and advice to aid them in managing their diabetes while they transition into college and adulthood. Currently, The Diabetes Link offers resources for students going off to college, caregivers, newly diagnosed individuals, and those starting their first full-time job [2].

While the organization offers valuable information, a component that is currently missing from their repertoire is nutrition. For the hundreds of millions of people in the United States not living with diabetes, mealtime is associated with feelings of family, excitement, and ease. However, for those with type-1 diabetes, each meal is met with feelings of stress, anxiety, and defeat. Despite nutrition being an important component to diabetes management, children with type-1 diabetes often do not meet dietary guidelines. Under some categories, they are less healthy than children without the diagnosis [3]. Current nutrition education for type-1 diabetes focuses on carbohydrate counting and general healthy eating, but it has no real teaching on food interactions and impacts on blood glucose levels [4]. While most people with type-1 diabetes know how to count carbohydrates to dose insulin accurately, most do not understand the impacts of other macronutrients, such as fats and proteins, on post-meal blood glucose level. Due to its chemical make-up, fat slows down the digestive process, causing an overall delayed reaction in glucose spikes [5]. While minor amounts of fat have little influence on overall blood sugar levels, an excess amount of fat in the diet has the potential to cause prolonged spikes in blood sugar levels. Like fat, protein is also slowly digested in the body. A balanced diet containing adequate amounts of fat, protein, and fiber can aid in slowing down carbohydrate digestion and delaying absorption and hence the possibility of post-meal spikes [5].

Despite the emphasis on accurate carbohydrate counting, research has shown that people with type-1 diabetes do not accurately count carbohydrates [6]. A trend highlighted in the same study showed that the longer a participant was living with diabetes, the less accurately they could count the carbohydrates of their meals. With the young adult population containing individuals that have been living with type-1 diabetes for 10, 15, or over 20 years, they fall within this category of frequent carbohydrate inaccuracies. In addition to this nutrition education gap, the young adult population that The Diabetes Link caters to is blatantly ignored in nutrition research. Most previous studies focused on children (those with diabetes under the age of 10), parents of children with type-1 diabetes, or people recently diagnosed but not the young adult population. This gap in subjects matters because most young adults do not meet A1C testing standards, a blood test that determines the average blood glucose level over the last three months [7]. Consistently high or uncontrolled blood glucose levels can have deadly complications, impacting major organs and causing nerve damage and blood vessel disease [8]. This study addresses some of these gaps by directly evaluating the population that the literature has previously failed to include. All the participants in this study reported an age between 18 and 30 years old. In addition, the distributed questionnaire evaluated the participants' health across several measures, including their nutrition literacy and emotional health.

The findings of this study were previously presented during Stony Brook University’s Thesis Day in April 2021.

## Materials and methods

Data were collected via a 40-question survey on Google Forms (Google LLC, Mountain View, California, United States). The survey encapsulated questions pertaining to the participants’ dietary habits, the emotional burden of the disease, treatments, and their confidence in healthcare staff to counsel them on nutrition in relation to diabetes. The survey also included four carbohydrate-counting questions to evaluate nutrition literacy. These questions stated a food item and portion size and asked participants in a multiple-choice manner how many carbohydrates they believed were in the item. The carbohydrate-counting questions were taken from a nutrition quiz found on the Juvenile Diabetes Research Foundation’s website and modified to fit the needs of the survey. All other survey questions were made to answer specific gaps encountered throughout the literature review. This portion of the questionnaire asked about patient care from biological, social, and psychological points of view. The survey was pilot-tested by volunteers from the organization to ensure clarity and accuracy before being distributed to the greater population.

The survey was distributed via The Diabetes Link’s emailing list every Friday for six weeks throughout January and February 2021. The organization also posted to its various social media accounts to inform followers of the survey link attached to the weekly newsletter to generate further interest. At the conclusion of the six weeks, 42 participants completed the surveys.

IBM SPSS Statistics for Windows, Version 27 (Released 2020; IBM Corp., Armonk, New York, United States) was utilized to analyze the collected data. To begin the analysis, ANOVA and chi-square tests were completed. Predictor variables included the Likert scale question pertaining to the burden level that the participants associated with eating with diabetes and the carbohydrate-counting quiz score. Dependent variables included if the participant was a part of a Diabetes Link chapter in college, if the participant ever avoided social gatherings because of food, if the participant ever skipped meals because of an out-of-range blood sugar level, and if they used a continuous glucose monitor as a part of their treatment. After conducting this analysis, a binary logistic regression analysis was performed to further investigate the above-mentioned variables. This study received IRB approval from Stony Brook University's Office of Research Compliance. 

## Results

The chi-square analysis shown in Table 1 highlights necessary demographic information and relevant health information, such as length of the participants' diagnosis. The chi-square analysis results featured in Table 1 show that the participants who reported higher levels of burden were less likely to be a part of a Diabetes Link chapter (p-value = 0.016) compared to those who were active members in their school's organization. The results in Table 1 also demonstrate that those who reported higher levels of burden associated with their diabetes also reported avoiding social gatherings because of food-related concerns.

**Table 1:** Summary of the demographics and burden level results of p-values based on the chi-square test

Level of perceived burden	1 (N=4)	2 (N= 4)	3 (N=16)	4 (N=12)	5 (N=6)	P-value
Female	3 (75%)	3 (75)	15 (93.75%)	9 (75%)	5 (83.3%)	0.630
Race						0.906
White	4 (100%)	4 (100%)	14 (87.5%)	12 (100%)	6 (100%)	
Black	0 (0%)	0 (0%)	1 (6.3%)	0 (0%)	0 (0%)	
Asian/Pacific Islander	0 (0%)	0 (0%)	1 (6.3%)	0 (0%)	0 (0%)	
Age						0.641
18-22	3 (75%)	2 (100%)	11 (68.75%)	6 (50%)	4 (66.6%)	
23-26	1 (25%)	1 (25%)	5 (31.25%)	5 (41.6%)	2 (33.3%)	
27-30	0 (0%)	1 (25%)	0 (0%)	1 (8.3%)	0 (0%)	
Length of diagnosis						0.992
0-5 years	1 (25%)	1 (25%)	5 (31.25%)	4 (33.3%)	2 (33.3%)	
6-10 years	1 (25%)	1 (25%)	3 (18.75%)	4 (33.3%)	2 (33.3%)	
11-15 years	1 (25%)	2 (50%)	6 (37.5%)	3 (25%)	1 (16.6%)	
15+ years	1 (25%)	0 (0%)	2 (12.5%)	1 (8.3%)	1 (16.6%)	
Was a part of a College Diabetes Network Chapter	1 (25%)	4 (100%)	14 (87.5%)	8 (66.6%)	2 (33.3%)	0.016
Avoided social gathering because of food	0 (0%)	0 (0%)	2 (12.5%)	3 (25%)	6 (100%)	0.002

The ANOVA analysis results highlighted in Table 2 show that the participants who scored higher on the carbohydrate-counting quiz (three or more questions correct) were more likely to utilize a continuous glucose monitor (p-value = 0.042) compared to those who scored lower (one or no questions correct on the quiz). Regarding the binary logistic regression highlighted in Table 3, the analysis showed that the participants who scored higher on the carbohydrate-counting quiz (three or more questions correct) were 2.389 times more likely to avoid eating because of an out-of-range blood sugar level compared to those who scored lower (one or no questions correct on the quiz). In addition, the participants who reported higher levels of burden due to the disease were 9.325 times more likely to avoid a social gathering because of food compared to those who reported lower levels of burden, as shown in Table 4.

**Table 2:** Summary of the carbohydrate-counting results p-values based on ANOVA

Carbohydrate-counting score					
Carbohydrate quiz performance	1 (N=8)	2 (N=12)	3 (N=12)	4 (N=10)	p-value
Currently use a continuous glucose monitor	8 (100%)	9 (75%)	12 (100%)	10 (100%)	0.042

**Table 3:** Summary of the carbohydrate-counting results of p-values based on the binary logistic regression model

Carbohydrate-counting score			
Carbohydrate quiz performance	S.E.	p-value	Exp(B)
Avoided eating because of an out-of-range blood sugar	.445	0.05	2.389

**Table 4:** Summary of the burden level results of p-values based on the binary logistic regression model

Burden level			
Level of perceived burden	S.E.	p-value	Exp(B)
Avoided social gathering because of food	.734	0.002	9.325

The burden level was self-reported via a Likert scale, with 1 correlating to no burden and 5 being highly burdensome. For all analyses regarding the burden level, the participants who reported lower burden (a score of 2 or less) were compared to those who reported high burden levels (4 or more). Other relevant findings include a mean carbohydrate-counting score of 2.57/4 and a mean reported HbA1c of 6.802%, which is comparable to a reading of 149 mg/dL. All HbA1c values were self-reported by the participants via the survey.

## Discussion

The findings indicate that the elevation of HbA1c levels in these ages might not necessarily be due to a lack of nutrition literacy but emotional burnout from the disease burden. As previously mentioned, most of the completed research in this field did not evaluate the demographic that this study specifically targeted. For example, a study evaluating the mode of nutrition education and its impacts on HbA1c levels was completed in a group of 151 poorly controlled diabetics. The results show that interactive patient-centered education was highly effective in improving the HbA1c levels in the participants, but the study focused only on those in the age group of 8-17 years. In addition, the study required the participants to be diagnosed with diabetes for only one year, while most young adults experiencing upticks in HbA1c levels have lived with their diagnosis most of their lives [9]. Other studies emphasized even younger ages, following children aged 7-14 years, when testing various methods of insulin dosing and nutrition education regarding HbA1c levels [10]. Similar to the previously mentioned research, this study also only focused on pediatric patients who were recently diagnosed. This demographic is highly different from the young adults who were diagnosed with type-1 diabetes that this pilot study utilized. In addition to most of the literature works utilizing younger, newly diagnosed patient populations, there is a huge lack of nutrition education research in the field overall. Most research on diabetes emphasizes looking for a cure or method to slow down or even possibly stop disease progression. For those already living with diabetes, studies focused on easing the burden of disease and possibility of complications that should be added to the plethora of current research. The results of this study demonstrate the need to create resources and aids to support diabetics in the long term and not just during the initial diagnosis stage. Investing in proper emotional aids can prevent some of the consequences of poor diabetes management, such as chronically elevated HbA1c levels.

Future research should look toward evaluating the mentality of diabetics when it comes to mealtime. The burden of disease is an emerging conversation in the diabetes sector as new technologies and innovations make healthy living with type-1 diabetes for decades feasible. This specific term of diabetes-related distress includes aspects of life, such as worrying about completing self-care tasks, feeling unsupported by family and care providers, and even feeling frustrated by success of care [11]. This idea of diabetes distress is difficult to pinpoint in populations that are recently diagnosed with diabetes or those too young to oversee most of their care. This point further stresses the need to broaden the patient populations used when conducting diabetes-related research.

This study also highlights the expansive influence that diabetes has on the biological, emotional, and social well-being of patients. The results from the survey show that diabetes is a multidimensional component in patients’ lives, and for the literature to reduce poor management down to nutrition solely is counterproductive to patient outcomes. The participant responses show that nutrition and diabetes impact multiple components of their lives, so treatment options and aids should be created and updated to solve these issues in a similar fashion. 

As previously mentioned, the amount of people being diagnosed and living with type-1 diabetes in the United States is on the rise, with some projections estimating that within the next 30 years, this number could reach five million [1]. The Diabetes Link is a non-profit organization with a vision to help this growing population through the most stressful times in life [12]. Working with experts in the field, they create informative and helpful resources, aids, and connections for young adults living with type-1 diabetes with the aim to make life a little easier. Such resources include fellowship programs, information on college-related activities like alcohol and diabetes, and the ins and outs of health insurance [13]. The Diabetes Link can be found on college campuses across the nation with almost 12,000 young adults impacted by the organization to date [12]. 

Key unexpected findings from this study pose the question of how to counter the unhealthy thoughts and behaviors developed by people with type-1 diabetes regarding their eating habits. Research on the relationship between young adults with type-1 diabetes and disordered eating is emerging, with results showing the need for clinicians to evaluate their patients with diabetes for disordered eating behavior during their yearly visits [14]. Future research should be directed toward how to properly screen young adult patients for these behaviors and how to aid those who do identify with disordered eating before diabetes-related complications arise.

A limitation to this study is the final sample size. Only 42 people completed the 40-question survey, calling into question the power of the study. It is also important to note that all information was self-reported. The type of treatment plan, past experiences, and education level of the healthcare professionals on the participants’ diabetes team might not be fully accurate because of the self-reporting aspect to the survey. Lastly, the target population could also be considered a limitation to the generalizability of the study. People who are members of The Diabetes Link have access to resources and support that the general public does not. This support could explain why reported HbA1c levels were below the national average for this age group and why the average carbohydrate-counting quiz score was at the passing level (65%). However, despite these limitations, the study has many strengths. It is the first study to encapsulate a wide range of questions, asking participants about multiple facets to their care and state of mind regarding their diagnosis. Questions on the survey asked about dietary habits, emotional outlook, and perception of healthcare provider understanding. It is also one of the first studies to specifically target young adults who have lived with diabetes for multiple years.

## Conclusions

Young adults living with type-1 diabetes report significantly higher HbA1c levels compared to other age groups. While most believe that this could be due to the lack of proper understanding on how nutrition can influence blood sugar, the results from this study demonstrate that this uptick might be due to disease burnout. Future points of care for this specific demographic of people with type-1 diabetes should emphasize biological, emotional, and social well-being in terms of disease management. The Diabetes Link works toward diversifying the aids and resources available to this population with their online workbooks, fellowship programs, and virtual events in efforts to provide holistic care to the community. This study clearly shows that this demographic is not necessarily lacking in nutrition knowledge. The participants scored well in the carbohydrate-counting quiz, with many obtaining perfect scores. Based on the results of this study, it is possible that this demographic of people with diabetes is in need of mental health support and resources to support them through stressful periods of life and times of disease burnout. Future research should further delve into this topic by evaluating the level of burden that patients perceive to have in terms of managing their diabetes and what resources and aids could assist in managing this fatigue. 
